# *In Vitro* Evidence of α‑Linolenic Acid Ethyl Ester Hydrolysis and Transesterification in the Gastrointestinal Tract in the Presence of Acylglycerols

**DOI:** 10.1021/acs.jafc.6c03597

**Published:** 2026-08-29

**Authors:** Gabriele Beltrame, Iida Valta, Eija Ahonen, Annelie Damerau, Kaisa M. Linderborg

**Affiliations:** † Food Sciences, Department of Life Technologies, University of Turku, FI-20014 Turku, Finland; ‡ Food and Nutrition Sciences, Department of Life Sciences (LIFE), Chalmers University of Technology, SE-41296 Gothenburg, Sweden

**Keywords:** ALA, ethyl ester, *in vitro* digestion, transesterification, bioavailability

## Abstract

Omega-3 polyunsaturated fatty acids (*n*-3 PUFA), such as α-linolenic acid (ALA), are crucial for human health. Although *n*-3 PUFAs in food are mainly in triacylglycerol (TAG) form, they are often provided as ethyl esters (EE) in supplements. Previous research has demonstrated improved postprandial bioavailability of *n*-3 PUFAs in the EE form when coingested with fat, potentially due to transesterification during digestion. To shed light on the bioavailability improvement, this study investigated the influence of triolein and 2-monoolein on the digestion of ALA-EE using an *in vitro* model, employing ^1^H NMR spectroscopy and liquid chromatography combined with mass spectrometry. ALA-EE hydrolysis increased from 6.6 to 22–29% with acylglycerols, reaching 55.8% after longer incubation with 2-monoolein. Transesterification of ALA in acylglycerols was observed, especially after incubation with 2-monoolein, but its correlation with ALA-EE hydrolysis remained ambiguous. Therefore, the bioavailability improvements were attributed to the facilitating effect of TAG lipolysis products on ALA release.

## Graphical abstract

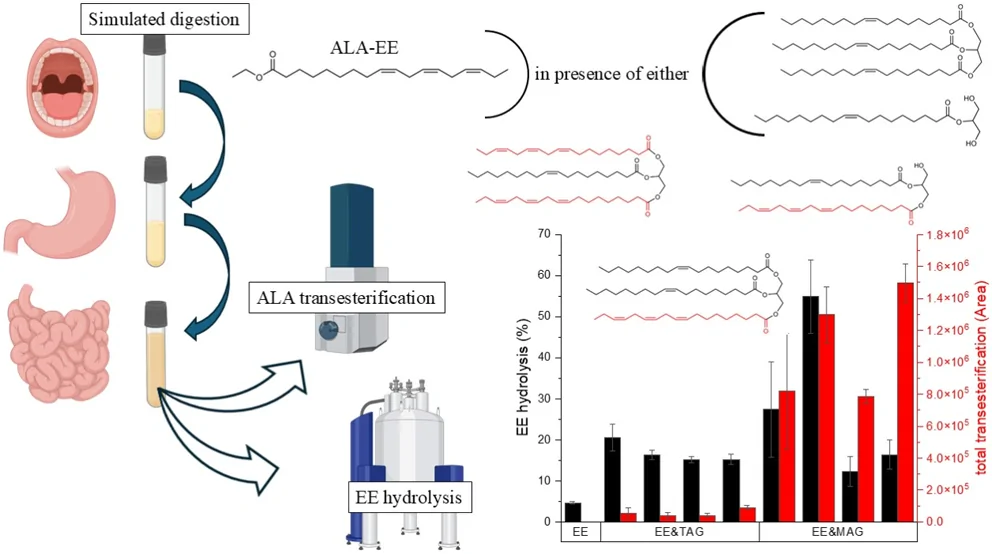

## 1 Introduction

Omega-3 polyunsaturated fatty acids (*n*-3 PUFAs) are important for the maintenance of human physiology. They are essential for the development of the brain and eye and play a role in the homeostasis of inflammation response, platelet aggregation, and vasodilation. They exert a homeostatic effect by counterbalancing the activity of omega-6 (*n*-6) PUFAs, which are involved, for example, in the triggering of inflammation and blood coagulation. Moreover, *n*-3 PUFAs provide protection against disorders, such as cardiovascular diseases.^1^

The *n*-3 PUFA α-linolenic acid (18:3*n*-3, ALA) is considered essential as it must be obtained through diet. Mammals can produce longer-chain PUFAs from ALA through a series of desaturation and elongation reactions. Specifically, ALA is converted into eicosapentaenoic acid (20:5*n*-3, EPA) and docosahexaenoic acid (22:6*n*-3, DHA).^2^ The European Food Safety Authority (EFSA) has recommended an adequate daily intake of *n*-3 PUFAs. The intake of ALA recommended by EFSA is 0.5% of dietary energy, while it is 250 mg per day for EPA plus DHA. Although 77% of the European population meets the ALA intake recommendations,^3^ the *n*-6 PUFA intake in Europe and America is reported to be 10-fold higher than ALA, with a high incidence of noncommunicable diseases, such as cardiovascular diseases and obesity, and consequent burden on large strata of the population and the healthcare system.^4^

Animal studies have suggested that maintaining the *n*-6/*n*-3 ratio below 4:1 leads to a tissue *n*-3 PUFA concentration that is beneficial to the human body.^5^ An intervention study showed that a daily intake of ALA can increase plasma EPA,^6^ while a meta-analysis linked flaxseed oil (>50% ALA w/w%) consumption to a reduction in blood inflammation markers.^7^ However, the desired intake of ALA-rich oils may exceed the recommended fat content. These issues can be addressed with the consumption of supplements, which can guarantee a daily intake of *n*-3 PUFA while keeping saturated fats low. The conversion of fatty acid esters from triacylglycerols (TAG) to ethyl esters (EEs) is a feasible method to produce supplements with a high *n*-3 PUFA content, as the EE of specific *n*-3 PUFAs can be distilled from the mixture.^8^

As TAGs are the original form of dietary *n*-3 PUFAs, concerns have long been raised about whether their bioavailability is maintained in the EE form. The release of *n*-3 PUFAs from the EE form has been investigated only twice using the consensus-based INFOGEST *in vitro* model of the gastrointestinal tract,^9^ and both studies suggested poor hydrolysis of EEs compared to TAGs.^10,11^ While different studies have shown lower plasma *n*-3 PUFA concentrations after EE consumption compared to TAGs,^12–14^ other clinical trials with long-term supplementation have concluded that the lipid structure has no role in the bioavailability of *n*-3 PUFAs.^15^ Moreover, previous studies have agreed that *n*-3 PUFAs in the EE form are better incorporated into plasma when they are coingested with fat.^16,17^ It has been hypothesized that the transesterification of *n*-3 PUFA in coingested acylglycerols could be responsible for the discrepancy between *in vitro* and *in vivo* data.^18^ On the other hand, novel delivery systems based on emulsification or encapsulation with other lipids have improved the *in vivo* bioavailability of *n*-3 PUFAs in the EE form.^13,14,19^

The present study aims to explain the enhanced bioavailability of *n*-3 PUFA-EE in the presence of fat by simulating the digestion of α-linolenic acid ethyl ester (ALA-EE) in the presence of acylglycerols utilizing the INFOGEST model. This study evaluated the hydrolysis of ALA-EE to estimate its bioavailability. The extent of ALA-EE hydrolysis is measured using ^1^H NMR spectroscopy and compared after digestion alone or in the presence of common dietary FA, oleic acid (18:1*n*-9), in the form of either TAG (triolein) or 2-MAG (2-monoolein). We hypothesize that the presence of acylglycerols (TAG and 2-MAG) would enhance the lipolysis of ALA-EE during simulated digestion, leading to a higher release of ALA compared to ALA-EE alone. The second objective of this study is to investigate ALA transesterification in the glycerol backbone of 2-monoolein or triolein during simulated digestion. The presence of transesterification products is verified using liquid chromatography-mass spectrometry. To the best of our knowledge, this is the first study to detail the *in vitro* bioavailability of *n*-3 PUFA-EE in the presence of fat using analytical methods.

## 2 Materials and Methods

### 2.1 Solvents, Reagents, and Digestion Fluid Components

Triolein (glyceryl trioleate, purity >99%), 2-monoolein (2-oleylglycerol, purity >95%), and α-linolenic acid ethyl ester (ALA-EE, purity >99%) were purchased from Larodan AB (Solna, Sweden). These compounds were dissolved in hexane and stored at −80 °C. Once dissolved, 0.05% α-tocopherol (Sigma-Aldrich, Saint Louis, MO) was added to the ALA-EE. For simulated digestion, α-amylase, pepsin, bile salts, and porcine pancreatin were purchased from Sigma-Aldrich (St. Louis, MO), while rabbit gastric lipase (RGE15-500) was supplied by Lipolytech (Marseille, France). All salts used for digestive fluids were obtained from VWR Chemicals (Leuven, Belgium). Potato flour was purchased from Finnamyl Oy (Kokemäki, Finland), whey protein concentrate from HSNG AB (Solna, Sweden), and wheat bran from Raisio Oy (Raisio, Finland). All chemical solvents were supplied by Sigma-Aldrich (Saint Louis, MO) unless otherwise specified. Ultrapure water was used for all work phases (Purelab Chorus instrument, Elga Veolia, High Wycombe, U.K.).

### 2.2 Simulated Digestion

#### 2.2.1 Lipid Component of Simulated Digestion

Simulated digestions were performed with ALA-EE together with varying amounts of triolein and 2-monoolein, and 2- or 4 h intestinal phase incubation. The ALA-EE/acylglycerol ratios used were 10:1 and 20:1 w/w^8^ for both acylglycerols. The amount of triolein was based on the production of 2-monoolein in simulated digestion (21% of total acylglycerols according to ^1^H NMR, see Section 2.3) to normalize the acylglycerol dosage. All the digestions were performed in triplicate. The design of the study is summarized in Supporting Table 1.

#### 2.2.2 Simulated Digestion Protocol

Simulated fluids for saliva, gastric, and small intestine were prepared according to the protocol.^9^ The pH of the simulated fluids was adjusted to 7.0 for the oral and intestinal phases, and to 3.0 for the gastric phase with 1 M HCl solution. The required aliquot of HCl was determined prior to digestion. The required aliquots of CaCl_2_·H_2_O (0.3 M) were added to the simulated fluids after the addition of enzymes, and the fluids were preheated at 37 °C. The whole procedure was performed under dim light, and digestion was carried out in the dark. All incubations were done at 37 °C in a linear shaking water bath (LAUDA, Lauda-Königshofen, Germany).

*In vitro* digestion started with a simulated oral phase, where 0.9 g of simulated defatted food was mixed with 40 mg of the lipid component^10^ for a final oil content of 10 mg/mL chyme. The bolus was added to 1 mL of simulated saliva containing 75 U/mL α-amylase. The mixture was randomly hit 32 times with a clean glass rod to simulate the chewing process, after which it was incubated for 2 min at 37 °C. In the simulated gastric fluid, the enzymatic activities of pepsin and rabbit gastric lipase were 2000 and 60 U/mL, respectively. Gastric fluid was added in a 1:1 ratio to the simulated oral bolus following 2 h of incubation at 37 °C. The simulated gastric chyme was added in a 1:1 ratio to the intestinal fluid and incubated for 2 or 4 h. In the simulated intestinal fluid, the concentration of bile salt was 10 mM. Porcine pancreatin was used to achieve 2000 U/mL lipase activity, as recommended by the INFOGEST protocol for studies focusing on lipid digestion.^9^

#### 2.2.3 Lipid Extraction

Lipids were extracted immediately after the digestion process. The samples were kept on ice during the whole procedure. Hexane/isopropanol (2:1 v/v) containing 0.05% w/v BHT (Sigma-Aldrich, Saint Louis, MO) was used as the extraction solvent. The extraction solvent was added to the digestates in a 1:1 v/v ratio, after which the tubes were vortexed and centrifuged at 3000 rpm for 3 min. The upper oil phase was collected, and the solvent was evaporated under nitrogen flow. The extraction was performed twice. The recovery of lipids was calculated gravimetrically. Samples were recovered with 3 mL of chloroform/methanol (1:1 v/v) and stored at −80 °C until further analysis.

### 2.3 ^1^H NMR Spectroscopy

Proton nuclear magnetic resonance spectroscopy (^1^H NMR) was used to analyze the hydrolysis of lipids. ^1^H NMR spectra were acquired for the digested and undigested ALA-EE samples. The lipid content of the samples was adjusted so that each sample contained a similar total concentration of ALA. The solvent was evaporated with nitrogen flow, and the lipids were recollected in 1 mL of CDCl_3_, which contained 0.03% tetramethylsilane (TMS) from Eurisotop (France). The samples were transferred to 5 mm NMR tubes with a syringe and stored at −20 °C in silica gel desiccators. The samples were prepared 24 h prior to the spectra recording. Each biological replicate was analyzed individually.

^1^H NMR spectra were recorded at 298 K and at a central frequency of 500.10 MHz with a Bruker Avance 500 (Bruker BioSpin Inc., Fällanden, Switzerland) equipped with Smartprobe, BB/1H, BCU I probehead cooling unit, and SampleCase automatic sample changer. Proton spectra (*zg*30) for each sample were collected with 32 scans and 2 dummy scans, a sweep width of 20 ppm, and a receiver gain of 128. The acquisition time was 3.28 s, and the relaxation time was 5 s. The length of the 90° shaped pulse was set automatically to 10 μs.^20^ NMR spectra were integrated and processed with TopSpin 4.0.7 (Bruker, MA). The TMS signal at 0.00 ppm was used as an internal standard to normalize integration results. For an accurate comparison of the signal areas among samples, the oscillations in concentrations among lipid digesta were corrected by multiplying each proton signal area by the ratio between the average area of ALA terminal −CH_3_ and the sample area of ALA terminal −CH_3_ (eq 1).

1 $$area\;{EE}^{*}=\frac{area\;{ALA-CH}_{3_{average}}}{area\;{ALA-CH}_{3_{sample}}}\times area\;EE$$

The hydrolysis of ALA-EE was estimated as a decrease in the area of the −CH_2_– signal after correction,^21^ following eq 2.

2 $$ALA‐EE\;hydrolysis\;\left(\%\right)=\frac{area\;{EE}_{undigested}^{*}-area\;{EE}_{digested}^{*}}{area\;{EE}_{undigested}^{*}}$$

On the other hand, the hydrolysis of triolein was estimated by considering the signal area of TAG relative to the sum of the TAG, 1,2-DAG, and 2-MAG signals from the ^1^H NMR spectra following eq 3.

3 $$TAG\;hydrolysis=1-\frac{A_{TAG}}{A_{TAG}+A_{1,2‐DAG}+A_{2‐MAG}}$$

### 2.4 UHPLC-ESI-QTOF

Ultrahigh-performance liquid chromatography combined with electrospray ionization and a quadrupole time-of-flight mass spectrometry (UHPLC-ESI-QTOF) was used for identifying the re-esterified compounds. Digested and undigested samples with 10:1 and 20:1 ALA-EE to triolein or 2-monoolein ratios were analyzed using Elute UHPLC and Bruker Impact II QTOF instruments (Bruker Daltonic, Bremen, Germany) and a Kinetex 2.6 μm PS C18 column (100 × 2.1 mm^2^) (Phenomenex, Torrance, CA). Each biological replicate was analyzed individually. The applied method was modified from our previous work.^10^ The temperature of the column oven was 30 °C, and that of the autosampler cooler was 4 °C. Samples were diluted to a concentration of 0.01 mg/mL in chloroform/methanol (1:1 v/v) and 1 μL was injected. Two mobile-phase solvents were used for analyte separation. Solvent A consisted of 50% water and 50% of methanol (Honeywell/Riedel de Haën, Seelze, Germany) and 10 mM ammonium formate (Sigma-Aldrich, Steinheim, Germany). Solvent B consisted of 70% 2-propanol (Honeywell/Riedel de Haën, Seelze, Germany), 30% methanol, 0.1% water, and 10 mM ammonium formate. The flow rate was 0.3 mL/min. The UHPLC gradient program started with 100% A solvent, followed by a linear increase of B solvent to 100% in 20 min, which was held for 5 min, and then decreased linearly to 0% in 2 min. The total run time was 30 min.

Electrospray ionization (ESI) was performed in the positive mode for mass spectrometry (MS). The ESI capillary and end plate offset voltages were set to 4.5 kV and 500 V, respectively. The nebulizer gas pressure was set to 1.5 bar, drying gas flow rate to 4 l/min, and drying gas temperature to 350 °C. Internal calibration was performed using sodium formate. Auto MS/MS scanning mode was tested for digested samples of triolein + ALA-EE with a mass range of 200–1200 *m*/*z* to identify the *m*/*z* values and retention times of the compounds of interest, which were used to determine the retention time windows for multiple reaction monitoring (MRM-MS) analysis. The retention time window of 8.5–15 min included MAGs and EEs, and the time window of 15–30 min included DAGs and TAGs. Bruker Compass DataAnalysis software (Bruker Daltonic, Bremen, Germany) was used for peak identification and peak area integration.

### 2.5 Statistical Analysis

Statistical analysis was performed with IBM SPSS Statistics 27 (IBM Corporation, Armonk, NY) after logarithmic transformation of the original data. The Shapiro–Wilk test was used to evaluate the normality of the data, and Welch and Brown–Forsythe were used as robust tests of equality of means. One-way analysis of variance (ANOVA) was performed using Levene’s test of homogeneity and Tukey or TamhaneT2 *post hoc* tests. Spearman’s rank correlations were computed for the variables. The statistical significance of differences among samples was considered at a confidence level of 95% (*p* < 0.05).

## 3 Results and Discussion

### 3.1 Identification of Digestion Compounds Using ^1^H NMR Spectroscopy

Proton nuclear magnetic resonance spectroscopy provides a complete molecular picture of lipolysis. The ^1^H signals of TAGs, DAGs, MAGs, and EEs were also identified. The assignments of the signals shown are reported in Supporting Table 2. The collected ^1^H NMR spectra of undigested ALA-EE and digested ALA-EE with and without triolein or 2-monoolein are shown in Figure 1. Enlarged selected regions showing TAG, DAG, and MAG signals with lower intensities are shown in Supporting Figure 1.

**Figure 1:**
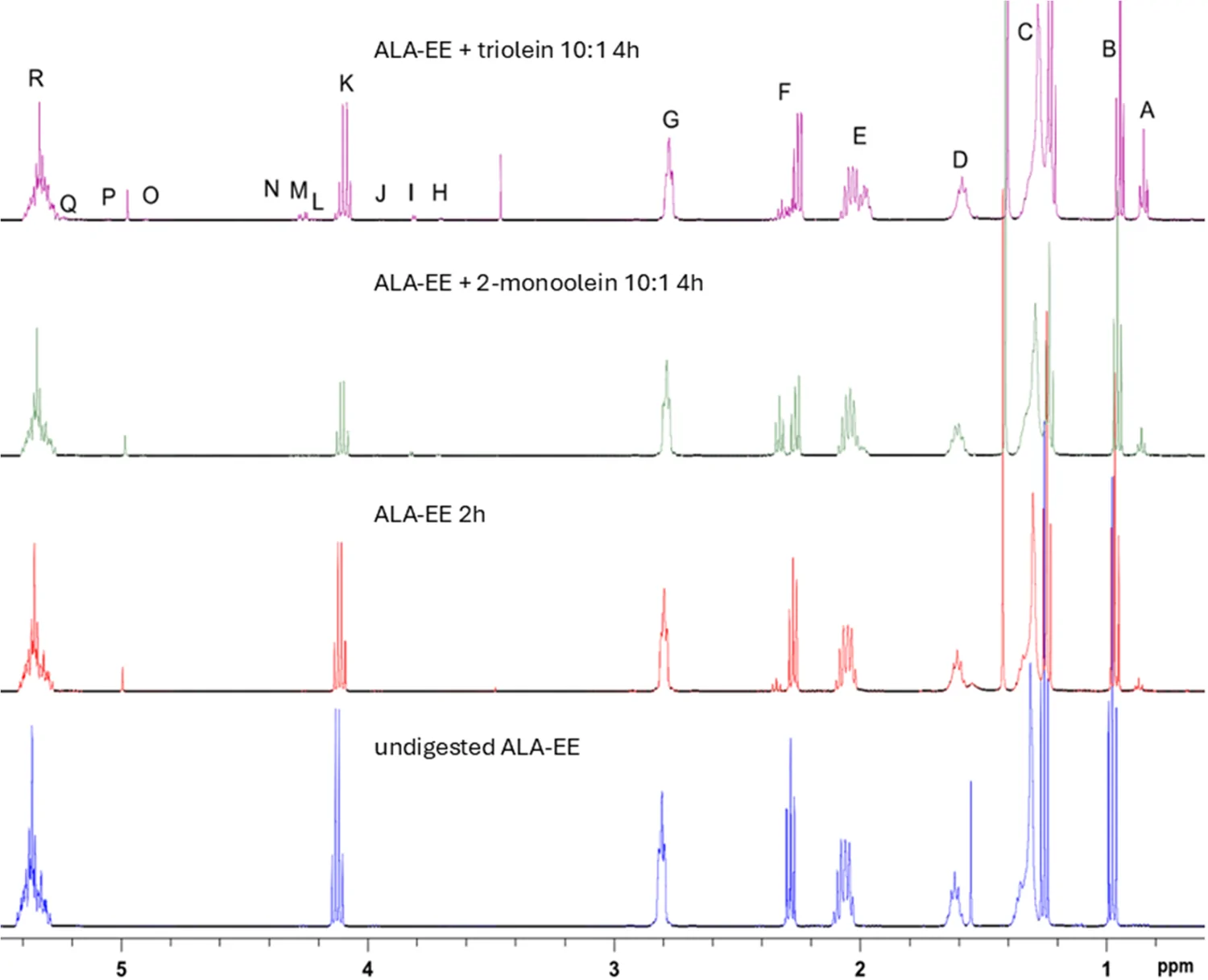
^1^H NMR spectra of lipid digesta and ALA-EE standard. Different letters represent the signal assignments listed in Supporting Table 2. Purple: ALA-EE + triolein 10:1, 4 h; green: ALA-EE + 2-monoolein 10:1, 4 h; red: ALA-EE alone 2 h; blue: undigested ALA-EE. The spectra were recorded at 298 K in CDCl_3_.

The terminal −CH_3_ signal of ALA was observed at 0.97 ppm, while samples containing acylglycerols of oleic acid showed a terminal −CH_3_ signal at 0.88 ppm. A small terminal −CH_3_ signal at 0.88 ppm was also observed in the digested ALA-EE sample, which was ascribed to the lipid content of the digestion matrix. The methylene signal of the ethyl ester (−CH_2_−) was a quartet at 4.12 ppm. Signals specific for different *sn*-positions of the glycerol backbone of TAGs were the multiplet at 5.26 ppm and two double doublets at 4.15 and 4.29 ppm. TAG signals were visible in the spectra collected from the triolein digesta, indicating incomplete hydrolysis. Noticeably, very low-intensity TAG signals were also present in the spectra of the digesta containing 2-monoolein (Supporting Figure 1). Signals specific to the glycerol backbone in 1,2-DAG were observed at 3.73, 4.33, and 5.08 ppm, and they were observed in samples containing triolein and 2-monoolein. Therefore, the recorded spectra showed the formation of new acylglycerols in the 2-monolein digesta. Signals for 2-MAG glycerol backbones were observed at 3.84 and 4.93 ppm in samples containing triolein and 2-monoolein, while the signals of 1-MAG were observed in all samples at 3.97 and 4.24 ppm. Pancreatin hydrolyzes ester bonds in both *sn*-1,3 positions, generating 1,2-DAG and 2-MAG, while rabbit gastric lipase has a higher affinity for position *sn-*3.^22^ The presence of 1-MAG was probably due to the acyl migration of 2-MAG from the *sn*-2 to *sn*-1/3 positions, as previously described.^23^ Acyl migration from the *sn*-2 to *sn*-1/3 positions has also been observed after prolonged intestinal phases of simulated digestion.^24^

### 3.2 Hydrolysis of ALA-EE

The present study utilized a semiquantitative approach. The ^1^H signals of EEs, TAGs, 1,2-DAGs, 2-MAGs, and 1-MAGs were integrated to evaluate the contents of different compounds in the undigested and digested samples. The signals of ethyl ester −CH_2_– at 4.12 ppm partially overlapped with those of TAG at 4.15 ppm, and therefore, their integration was performed very carefully.

According to our current understanding of EE digestion, hydrolysis into free fatty acids (FFAs) is a required step for their incorporation into mixed micelles and absorption by intestinal enterocytes.^18^ Therefore, this study evaluated the hydrolysis of ALA-EE to estimate its bioavailability. Hydrolysis was expressed as a decrease of the ^1^H NMR signal of ALA-EE −CH_2_– at 4.12 ppm after simulated digestion. The results are shown in Figure 2. In the absence of acylglycerols, the hydrolysis of ALA-EE was 6.6 ± 0.2%. This low value was in accordance with previous studies, reporting *n*-3 PUFA-EE hydrolysis at or below 10% in simulated digestion.^10,25–27^

**Figure 2:**
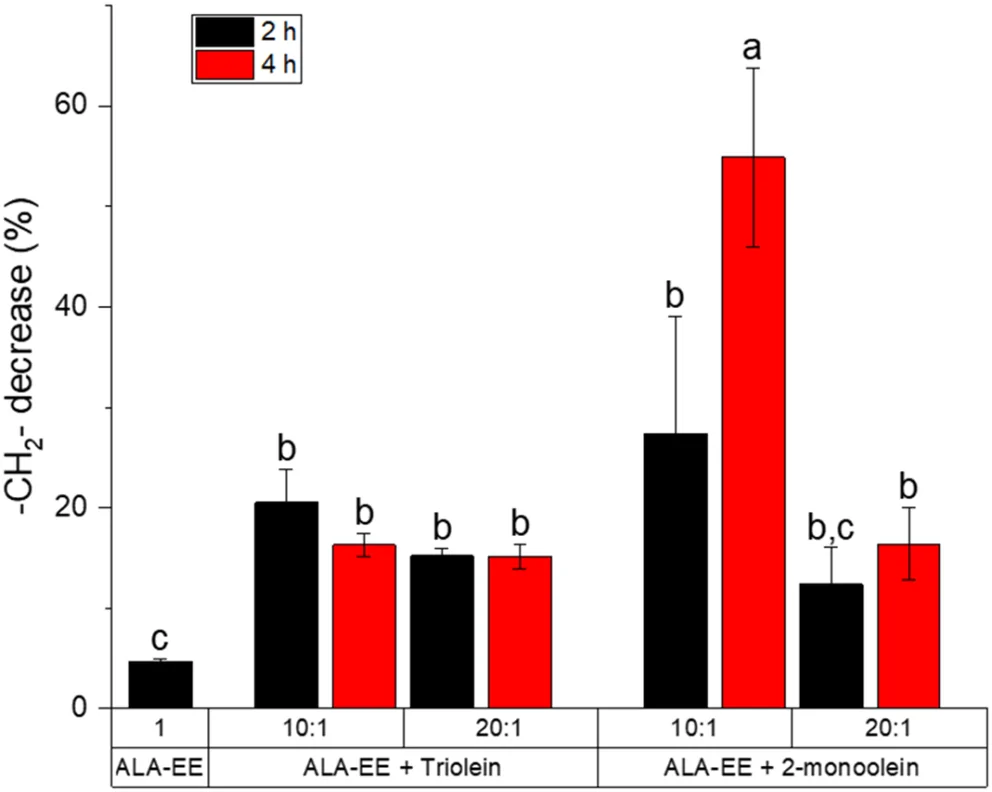
Hydrolysis of α-linolenic acid ethyl ester (ALA-EE) after simulated digestion is represented by a decrease of the ALA-EE methylene (−CH_2_−) signal in the ^1^H NMR spectra. Values are average ± standard deviation (*n* = 3). Different letters mark statistically significant (*p* < 0.05) differences.

Hydrolysis of ALA-EE increased significantly (*p* < 0.05) when digestion was performed in the presence of triolein (Figure 2). After 2 h of intestinal phase, the hydrolysis of ALA-EE was 22.1 ± 2.7 and 16.9 ± 0.6% in samples with 10:1 and 20:1 ALA-EE/triolein ratios, respectively. However, the ALA-EE/triolein ratio had no statistically significant effect on hydrolysis. Also, increasing incubation time during the intestinal phase had no significant effect (*p* > 0.05) on ALA-EE hydrolysis (Figure 2). By contrast, Yang et al.^27^ observed that the presence of triolein was detrimental to the hydrolysis of ethyl oleate. However, the grouping of the two data sets (Supporting Figure 2) showed that this was more likely due to different EE/triolein ratios. The hydrolysis of triolein was also estimated by considering the signal area of TAG in relation to the sum of the TAG, 1,2-DAG, and 2-MAG signals from the ^1^H NMR spectra. The results are shown in Supporting Figure 3. For samples with a 10:1 ratio, the hydrolysis of triolein was 28.9 ± 2.3 and 31.9 ± 2.6% after 2 and 4 h, respectively. These values were statistically similar (*p* > 0.05) to those of triolein digested alone. For samples with a 20:1 ratio, the hydrolysis of triolein was 46.3 ± 4.8 and 55.1 ± 2.6% after 2 and 4 h, respectively, which were significantly different from each other and from the 10:1 ALA-EE/triolein samples. The increase in triolein lipolysis can be attributed to its decrease in the absolute amount, a relationship inferred from the previous literature^28,29^ and also observed in our lab.^30,31^

The highest hydrolysis of ALA-EE (55.8 ± 7.1%) was achieved when digestion was performed in the presence of 2-monoolein at a 10:1 ALA-EE/2-monoolein ratio and when the intestinal phase was extended to 4 h. This result was statistically significantly higher (*p* < 0.05) than the others (Figure 2). The standard 2 h intestinal phase, which resulted in 28.8 ± 9.4% hydrolysis, did not differ significantly from the triolein incubations. Moreover, no considerable increase of hydrolysis was observed compared with triolein when ALA-EE was supplemented with 2-monoolein at a 20:1 ratio at either incubation time (Figure 2).

The higher hydrolysis of ALA-EE in the presence of 2-monoolein compared to triolein can be explained by the absence of hydrolyzable ester bonds in 2-monoolein, while the *sn*-1/3 positions of triolein are substrates for hydrolysis, which is the favored reaction in an aqueous medium.^32^ At the same time, the surface activity of 2-monoolein could have aided in the emulsification and the hydrolysis of the ethyl ester bond. The formation of emulsions during digestion is necessary for the anchoring of pancreatic lipase, as it acts at the lipid–water interface.^32,33^

In the Yang data set, the rate of hydrolysis of oleic acid in the TAG form was about 1.8 times faster than that of ALA-EE and about the same as that of ALA (1.5 and 1.3, respectively) in the TAG form. The authors concluded that EEs were unable to compete with TAGs for occupancy of the lipid droplet surface due to their lower polarity and thus greater solubility in the hydrophobic interior of lipid droplets.^27^ Our previous results^21,31^ hinted at a slight preference of pancreatic lipase for *n*-3 and *n*-6 rather than *n*-9 FA, but the differences had little significance. The difference in hydrolysis rates has also been studied by Dalheim, who showed that PUFAs were released more slowly *in vitro* than MUFAs in both TAG and EE forms.^25^ Overall, pancreatic lipase favors the hydrolysis of C18 esters in TAG over EE. Pancreatic lipase may have a lower affinity for EEs during simulated digestion, possibly due to the evolutionary specialization of the enzyme for acylglycerols, as the hydrophobic pocket accommodates the bulky Y-shaped structure of TAGs.^34^ At the same time, differences in the emulsification of EE compared to TAG during digestion have also been considered.^27^

According to the literature, although the hydrolysis of EEs is slower than that of TAGs, esterified *n*-3 PUFAs eventually reach a similar extent of absorption *in vivo*. Yang fed menhaden oil or the corresponding EEs to rats, and the FFA in prechylomicrons were overall similar, except for 18:4*n*-3 and 20:4*n*-6, which had higher relative amounts after EE consumption, and DHA, which had a higher relative amount after oil consumption. Similarly, the amounts of free ALA in prechylomicrons had no substantial difference when administered as rapeseed oil or methyl ester.^35^ In another study, when EPA was fed to rats as EE, its blood concentration was lower compared with that of the 2-monoeicosapentaenoin form. When the formulas were subjected to simulated digestion, lipolysis of EPA-EE was lower than that of 2-monoeicosapentaenoin, but only slightly lower than that of trieicosapentaenoin.^36^ In a human trial, West observed little differences in the median blood concentrations of EPA and DHA after consumption of PUFA-rich oil or the corresponding EEs, but these were consumed together with an emulsion of olive and flaxseed oils in large excess compared to the PUFA dosage.^15^ A recent review on PUFA bioavailability noted that chronic supplementation studies tend to show higher bioavailability of PUFAs in the EE form compared to acute studies.^12^

The discrepancy between our results and those of previous studies could also partly be ascribed to the limitations of the digestion model. INFOGEST is a static digestion model, and the absence of the removal of digestion products from the simulated chyme could disfavor hydrolysis. The amount of pancreatin was also static, while *in vivo* pancreatic lipase secretion increases with the amount of fat present in the meal.^37^ Further, bile salts were used to solubilize the digestion products into mixed micelles, but their concentration, although following the standardized protocol, could have been suboptimal for the present study.^24^ Moreover, INFOGEST does not recommend a shear force to be applied to the chyme to simulate peristalsis. Previous work has observed that higher shear increases oil lipolysis.^28^

### 3.3 Identification of Transesterification Products with UHPLC-ESI-QTOF

Lipid species from undigested samples and digestates were identified with UHPLC-ESI-QTOF. Multiple reaction monitoring (MRM-MS) was used in this study to detect the fragmentation of the specified precursor masses. It provides higher sensitivity and specificity than auto-MS/MS, where precursor ions are automatically selected from those with the highest abundance. The approach has clear benefits for the analysis of complex matrices such as lipid digestates, where possible matrix effects and coeluting compounds may compromise analyte identification and quantification.^38^

The *m*/*z* values of the ammonium and sodium adducts of the identified compounds are presented in Table 1. Details for the identification of undigested lipids and digestion compounds are reported in Supporting Text. For simplicity, the incorporation of ALA into both triolein and 2-monoolein is referred to as a transesterification reaction. The identified compounds resulting from the transesterification between ALA and 2-monoolein or triolein were DAG 18:3-18:1, TAG 18:3-18:1-18:1, and TAG 18:3-18:1-18:3. We also observed weak ion currents for the TAGs 18:1-18:2-18:2 and 18:2-18:2-18:3, which are most likely residues from the synthesis of pure triolein and 2-monoolein. Because of the pancreatic lipase specificity for *sn*-1/3 positions, the absence of 18:1 in *sn*-1/3 positions would be expected when ALA-EE was digested with 2-monoolein. Therefore, the presence of TAG 18:3-18:1-18:1 in the samples digested with 2-monoolein can be explained by the acyl migration of C18:1. It is known that acyl migration increases with longer reaction times.^8^ On the other hand, TAG 18:3-18:1-18:3 was absent in samples digested with triolein, probably because hydrolysis of triolein was not complete and there was still 1,2-DAG present after the 4 h intestinal phase (Supporting Figure 1). As mentioned earlier, pancreatic lipase tends to prefer the hydrolysis and esterification of oleic acid over ALA.^27^ The double bonds in ALA cause steric hindrance, hampering the esterification of a second ALA molecule by lipases. This also explains the relatively high amounts of DAG 18:3-18:1 in the digestates with 2-monoolein. It has been previously shown that the incorporation of one molecule of EPA into tricaprylin was faster than the incorporation of a second EPA molecule.^39^

**Table 1:** Compounds and Their Ammonium [M + NH_4_]^+^ and Sodium [M + Na]^+^ Adduct Masses (*m/z*) from UHPLC-ESI-QTOF Chromatograms, along with Retention Times and Fragments Used for Identification (Mass Values are in Bold)

compound	*m*/*z*	[M + NH_4_]^+^	[M + Na]^+^	RT (min)	main fragments^a^
MAG 18:1	356.293	**374.327**	379.284	13.2	339.291, 265.255, 247.244
EE 18:3	306.256	**324.291**	329.247	14.9	307.265, 261.223, 243.213
DAG 18:3-18:1	616.507	**634.541**	639.500	18.3	617.517, 599.507, 339.290, 335.260
DAG 18:1-18:1	620.538	**638.577**	643.531	19.1	621.550, 603.538, 339.292, 321.272
TAG 18:3-18:1-18:3	876.721	**894.760**	899.711	21.0	877.730, 859.786, 599.507, 595.479, 339.292, 335.260
TAG 18:3-18:1-18:1	880.752	**898.787**	903.747	21.2	881.765, 863.8, 603.539, 599.507, 339.292, 335.257
TAG 18:1-18:1-18:1	884.783	**902.824**	907.780	21.5	885.798, 603.538, 339.291
TAG 18:1-18:2-18:2	880.752	**898.787**	903.752	21.2	881.770, 601.519, 599.507
TAG 18:2-18:2-18:3	876.721	**894.762**	899.717	21.0	877.733, 599.507, 597.490

a Details of the identification of undigested lipids and digestion compounds are reported in Supporting Text.

Compound peak area data were obtained from the extracted ion chromatograms, and the extent of transesterification was evaluated based on the area of the transesterification products. The integrated peak areas of the identified transesterification products DAG 18:3-18:1, TAG 18:3-18:1-18:1, and TAG 18:3-18:1-18:3 are presented in Figure 3. When triolein was digested with ALA-EE, only small amounts of ALA were transesterified either at the *sn*-1 or *sn*-3 position of triolein. In contrast, when ALA-EE was digested with 2-monoolein, a higher amount of ALA was incorporated into the acylglycerols. Prolonging the intestinal phase resulted in higher levels of transesterification in samples containing 2-monoolein. The peak area of TAG 18:3-18:1-18:1 was significantly (*p* < 0.05) higher in the sample with a 10:1 ALA-EE to 2-monoolein ratio and 4 h intestinal phase compared to all samples digested with triolein, except for the sample with a 10:1 ALA-EE to triolein ratio and 2 h intestinal phase. The most abundant transesterification compound present in the samples digested with 2-monoolein was TAG 18:3-18:1-18:3. The peak area of TAG 18:3-18:1-18:3 was significantly higher (*p* < 0.05) after 4 h compared to the 2 h intestinal phase in samples with a 20:1 ratio. A similar trend was observed for the samples with a 10:1 ratio, despite the lack of a significant difference. Also, peak areas of DAG 18:3-18:1 increased after the 4 h intestinal phase, but there were no statistically significant differences between 2 and 4 h.

**Figure 3:**
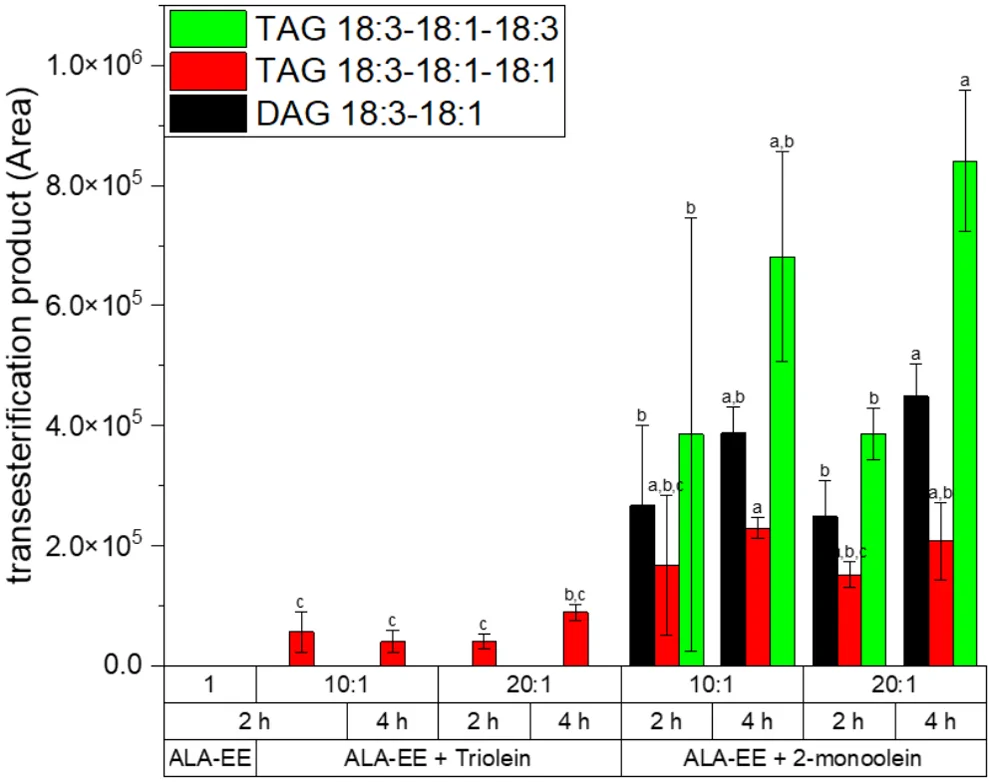
Peak areas of compounds formed during transesterification between ALA and triolein or 2-monoolein after INFOGEST *in vitro* digestion. The peak areas are obtained from the UHPLC-ESI-QTOF ion currents outlined in Table 1. Values are expressed as average ± standard deviation (*n* = 3). Different letters indicate statistically significant (*p* < 0.05) differences.

The ratio between the sum of the transesterification products and 2-monoolein was calculated from both the mass spectrometry and ^1^H NMR data for the samples digested with 2-monoolein (Supporting Table 3). In the case of 2-monoolein, it can be assumed that the TAG and DAG signals from the ^1^H NMR spectra represented the transesterification products. The purity of 2-monoolein was over 95% according to the manufacturer, and mass spectrometric data confirmed that DAGs with two oleic acids (18:1-18:1) were absent in the digestates obtained from 2-monoolein. Both mass spectrometry and ^1^H NMR spectroscopy showed the same trend, i.e., a higher ratio of transesterification products to 2-monoolein when the intestinal phase was 4 h. However, the extension of transesterification can only be estimated from our results, as no standards were used. Moreover, the ionizability of molecules in digestates could have been influenced by salt residues from digestive fluids.^10^ On the other hand, ^1^H NMR may underestimate TAG formation due to the overlapping between the methylene moiety of ethyl ester and TAG signals.

The promotion of transesterification by 2-monoolein can be explained by multiple factors. Both lipase and the substrates for the transesterification reactions are located at the oil–water interface of emulsified lipids in the chyme.^40^ The supplementation of 2-monoolein implies that it is readily available to act as a substrate for lipases, whereas triolein requires hydrolysis. In addition, the adsorption of lipase at the oil–water interface may have been aided by the emulsification activity of 2-monoolein.^32^ Moreover, conformational changes caused by the interaction with 2-monoolein could improve the catalytic activity of lipases.^33^

A possible explanation for the limited transesterification of ALA with triolein and 2-monoolein is the high water content present in the simulated gastrointestinal tract. In their study, Bogevik and co-workers investigated the enzymatic interesterification of heterotrophic microalgae oil and rapeseed oil. They observed that higher amounts of re-esterified TAGs were formed when water was absent in the reaction medium. Conversely, moisture directed the reaction toward hydrolysis and led to higher amounts of FFAs in comparison with the absence of water.^41^ Moreover, reaction times longer than 4 h may be required to achieve the maximum yield of transesterification products, as shown in many studies.^8,39,41^ However, these reaction times would represent unrealistic digestion times. At the same time, in the work of Castejón and Señoráns, when EPA/DHA-EE was transesterified with 2-MAG, only slightly higher yields of structured TAGs were achieved after 24 h compared to 4 h.^8^ In addition, Wu and co-workers showed that, after a 24 h reaction time at 37 °C, porcine pancreatic lipase esterified 50% of free oleic acid with 1-butanol, while only 19% of rapeseed oil fatty acids were esterified with 2-ethyl-1-hexanol.^42^ They concluded that the hydrolytic activity of the enzyme had no necessary correlation with esterification activity.^42^

### 3.4 Correlation between Hydrolysis and Transesterification of ALA-EE

The total area of the transesterification products in relation to the degree of hydrolysis of ALA-EE is shown in Figure 4. In addition, the same relationship with the area of the single transesterification products identified in this work is shown in Supporting Figure 4.

**Figure 4:**
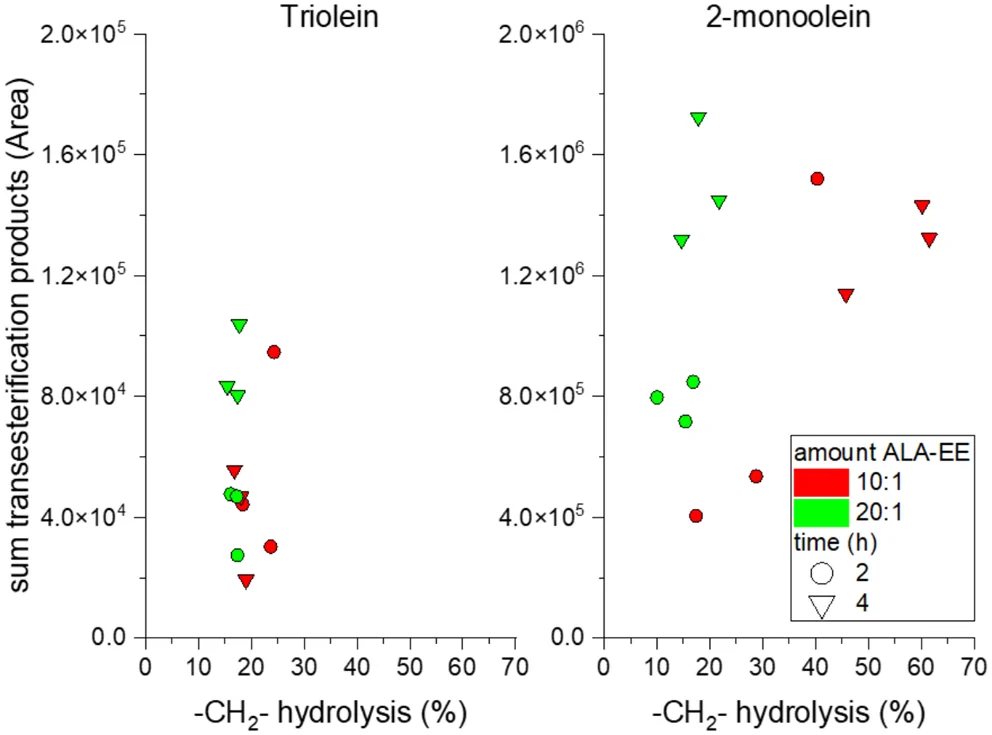
Scattering plot showing the relationship between the sum of UHPLC-ESI-QTOF peak areas of transesterification products and hydrolysis of ALA-EE analyzed using ^1^H NMR spectra in the respective digesta.

Figure 4 shows a correlation between the transesterification and hydrolysis. Such a correlation would be expected, as the identified transesterification products are in equilibrium with free ALA. However, the aggregated data obtained with 2-MAG showed a positive but insignificant correlation between ALA-EE hydrolysis and the sum of the transesterification product areas (ρ = 0.40, *p* > 0.05). Lack of significance was also observed with the disaggregated data set, i.e., at a 10:1 ratio (ρ = 0.60, *p* > 0.05) and at a 20:1 ratio (ρ = 0.66, *p* > 0.05). Our results clearly indicated that acylglycerol species are the driving force for transesterification, with no clear effect of other digestion conditions on transesterification. As shown in Figures 3, 4, and Supporting Figure 4, the increase in intestinal incubation time, rather than ALA-EE content, was responsible for the increased transesterification. Therefore, mass action in hydrolysis equilibria fails to fully explain transesterification. This indicates that the insertion of ALA into acylglycerols was rather limited, and most of the released ALA remained in the FFA form. This can be explained by the preferential removal of FFAs from the oil–water interface and their incorporation into mixed micelles by bile salts.

In conclusion, the objective of this study was to investigate the gastrointestinal behavior of ALA-EE using the INFOGEST simulated digestion model. Hydrolysis of ALA-EE was very low in the absence of other dietary fats but increased significantly when the digestion mixture contained acylglycerols. To the best of our knowledge, the bioavailability of *n*-3 PUFA EEs in the presence of dietary TAGs was analyzed using the INFOGEST protocol for the first time. Our results are consistent with those of clinical trials claiming that fatty meals improve the bioavailability of EEs. Higher relative amounts of EEs may have restricted lipolysis due to the reduced presence of digestion products with emulsifying properties. This study confirmed previous evidence that the hydrolysis of TAGs is favored over EE. In addition, the present findings are consistent with the enhanced *n*-3 PUFAs EE bioavailability observed in clinical trials with formulations based on lipid emulsifiers.^14,43^ Although promising, the commercialization of *n*-3 PUFA EEs supplemented with lipid emulsifiers remains limited by regulatory and standardization challenges. The methodology used in this study could serve as a valuable tool for screening and comparing formulations prior to *in vivo* evaluation.

The present study investigated whether ALA-EE could be transesterified with triolein or 2-monoolein in a simulated gastrointestinal tract to enhance *n*-3 PUFA bioavailability. Three different transesterification products (DAG 18:3-18:1, TAG 18:1-18:1-18:3, and TAG 18:3-18:1-18:3) were identified in digestates using UHPLC-ESI-QTOF. Greater extents of ALA incorporation in 2-monoolein were observed at a 10:1 ratio after a 4 h intestinal phase. In contrast, only minor amounts of ALA were transesterified in the acylglycerols originating from triolein. The *in vitro* results indicated that the transesterification of ALA into the glycerol backbone in simulated intestinal contents has little relevance for the higher bioavailability of EEs in the presence of TAGs observed *in vivo*. Instead, it can be concluded that *n*-3 PUFA EEs are hydrolyzed into FFAs, with acylglycerols aiding in EE emulsification and FFA solubilization to form mixed micelles.

## Supplementary Material



## Notes

Magnification of ^1^H NMR spectra on the integrated regions (Supporting Figure 1); comparison of the present data set with that of Yang et al. on EE hydrolysis in the presence of TAGs (Supporting Figure 2); degree of hydrolysis of triolein (Supporting Figure 3); relationship between UHPLC-ESI-QTOF peak areas of single transesterification products and hydrolysis of ALA-EE (Supporting Figure 4); summary of the different experimental conditions used in this study (Supporting Table 1); chemical shift assignments and multiplicities of the ^1^H NMR signals used in this study (Supporting Table 2); ratios between the sum of transesterification products and 2-monoolein after simulated digestion (Supporting Table 3) (PDF)

## Abbreviations

^1^H NMR: proton nuclear magnetic resonance spectroscopy
ALA: α-linolenic acid (18:3*n*-3)
ANOVA: analysis of variance
DAG: diacylglycerol
DHA: docosahexaenoic acid (22:6*n*-3)
EE: ethyl ester
EFSA: European Food Safety Authority
EPA: eicosapentaenoic acid (20:5*n*-3)
FA: fatty acid
FFA: free fatty acid
MAG: monoacylglycerol
MRM: multiple reaction monitoring
*n*-3 PUFA: omega-3 polyunsaturated fatty acid
TAG: triacylglycerol
UHPLC-ESI-QTOF: ultrahigh-performance liquid chromatography combined with electrospray ionization and quadrupole time-of-flight mass spectrometry
