# Supplementary material for: In Vitro Evidence of α‑Linolenic Acid Ethyl Ester Hydrolysis and Transesterification in the Gastrointestinal Tract in the Presence of Acylglycerols
Source: J Agric Food Chem. 2026 Aug 29;74(35):27512–20. doi: 10.1021/acs.jafc.6c03597 (PMC13573556; doi:10.1021/acs.jafc.6c03597)
Supplement: Supplementary file 1 [file jf6c03597_si_001.pdf]

Supporting information

In vitro evidence of α-linolenic acid ethyl ester hydrolysis and transesterification in the presence of acylglycerols in the gastrointestinal tract

Gabriele Beltrame[†,ⱡ,‡], Iida Valta[†,‡], Eija Ahonen[†], Annelie Damerau[†], Kaisa M. Linderborg[†,*]

[†]Food Sciences, Department of Life Technologies, University of Turku, FI-20014 Turku, Finland

[ⱡ]Food and Nutrition Sciences, Department of Life Sciences (LIFE), Chalmers University of Technology, SE-41296 Gothenburg, Sweden

[‡]These authors contributed to the work equally

*Corresponding author:

Professor Kaisa Linderborg

Food Sciences Unit, Department of Life Technologies

University of Turku, FI-20014 Turku, Finland

email: kaisa.linderborg@utu.fi; phone: + 358 50 439 5535

Supplementary Text. Identification of undigested compounds and digestion products.

The product ion spectra of the investigated glycerolipids and fatty acid ethyl ester showed fragmentation pathways characteristic of their respective lipid classes. For MAG 18:1, the ammoniated precursor ion ($[M+NH_4]^+$, *m/z* 374.327) formed fragments corresponding to the loss of water from the $[M+H]^+$ precursor (*m/z* 339.291), to the 18:1 acyl chain (*m/z* 265.255), and to the loss of water from the 18:1 acyl chain (*m/z* 247.242). The ammoniated ALA-EE ($[M+NH_4]^+$, *m/z* 324.291) initially underwent loss of ammonia to produce *m/z* 307.265 ($[M+H]^+$). Subsequent elimination of ethanol yielded the fragment at *m/z* 261.223 ($[M+H-C_2H_6O]^+$), while the ion at *m/z* 243.213 was tentatively assigned to further dehydration of the acyl-derived fragment. With triolein (TAG 18:1-18:1-18:1) ($[M+NH_4]^+$, *m/z* 902.824), loss of ammonia generated the protonated molecular ion at *m/z* 885.798, while cleavage of any ester bond produced a single DAG 18:1-18:1 fragment at *m/z* 603.538, which further fragmented to the protonated MAG 18:1 ion at *m/z* 339.291.

Among the molecules identified in digesta, both DAG 18:3-18:1 and DAG 18:1-18:1 exhibited the characteristic fragmentation behavior of ammoniated DAGs. For DAG 18:3-18:1 ($[M+NH_4]^+$, *m/z* 634.541), the major fragments included the protonated molecule at *m/z* 617.517 ($[M+H]^+$) and its dehydrated product at *m/z* 599.507 ($[M+H-H_2O]^+$). Cleavage of the glycerol backbone produced protonated fragments at *m/z* 339.290 and 335.260, corresponding to ionized MAG 18:1 and MAG 18:3. Similarly, DAG 18:1-18:1 ($[M+NH_4]^+$, *m/z* 638.577) generated the protonated ion at *m/z* 621.550 and the dehydrated ion at *m/z* 603.538, together with the protonated MAG 18:1 fragment at *m/z* 339.292 and its dehydration product at *m/z* 321.272.

Among the identified TAGs in digesta, TAG 18:3-18:1-18:3 ($[M+NH_4]^+$, *m/z* 894.760) produced the protonated molecular ion at *m/z* 877.730 and the dehydrated ion at *m/z* 859.786. Neutral loss of an 18:3 fatty acid yielded the DAG 18:1-18:3 fragment at *m/z* 599.507, whereas

loss of the 18:1 chain generated the DAG 18:3-18:3 fragment at *m/z* 595.479. Further fragmentation produced protonated MAG 18:1 and MAG 18:3 ions at *m/z* 339.292 and 335.260, respectively. TAG 18:3-18:1-18:1 ($[M+NH_4]^+$, *m/z* 898.787) exhibited analogous fragmentation, giving the protonated molecular ion (*m/z* 881.765) and its dehydration product (*m/z* 863.800). The ion at *m/z* 603.539 corresponded to the protonated DAG 18:1-18:1 fragment produced after loss of the 18:3 fatty acid, while the ion at *m/z* 599.507 represented the DAG 18:3-18:1 fragment formed after loss of an 18:1 chain. Subsequent fragmentation generated the MAG 18:1 and MAG 18:3 ions at *m/z* 339.292 and 335.257, respectively. The minor TAG 18:1-18:2-18:2 ($[M+NH_4]^+$, m/z 898.787) yielded the protonated molecular ion at *m/z* 881.770. The ion at *m/z* 601.519 corresponded to DAG 18:1-18:2, produced by neutral loss of an 18:2 fatty acid, whereas the ion at *m/z* 599.507 represented DAG 18:2-18:2, formed after loss of the 18:1 chain. Similarly, TAG 18:2-18:2-18:3 ($[M+NH_4]^+$, *m/z* 894.762) generated the protonated precursor at *m/z* 877.733. Cleavage of the ester bonds produced the diagnostic DAG fragments at *m/z* 599.507, corresponding to DAG 18:2-18:2, and *m/z* 597.490, corresponding to DAG 18:2-18:3.

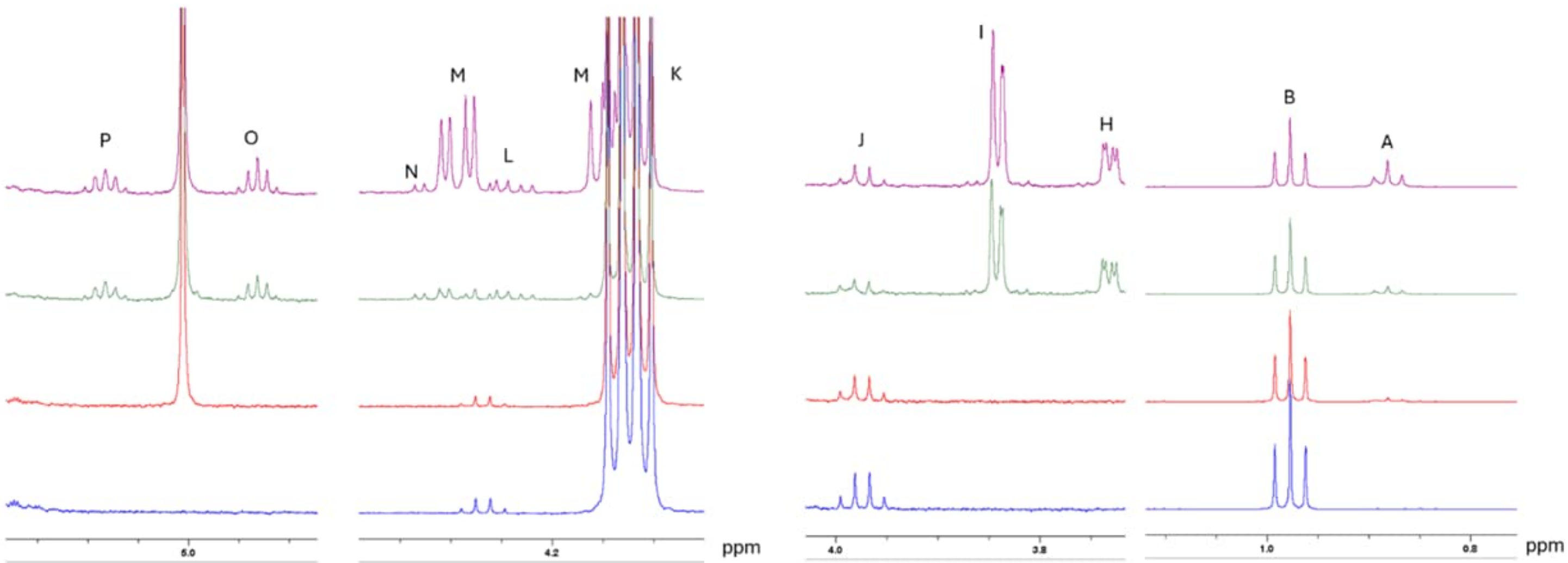

Supplementary Figure 1. Spectral regions at different magnifications selected to highlight the integrated signals. Different letters represent signal assignments disclosed in Supplementary Table 2. Purple: ALA-EE + triolein 10:1 4h; green: ALA-EE + 2-monoolein 10:1 4h; red: ALA-EE 2h; blue: undigested ALA-EE.

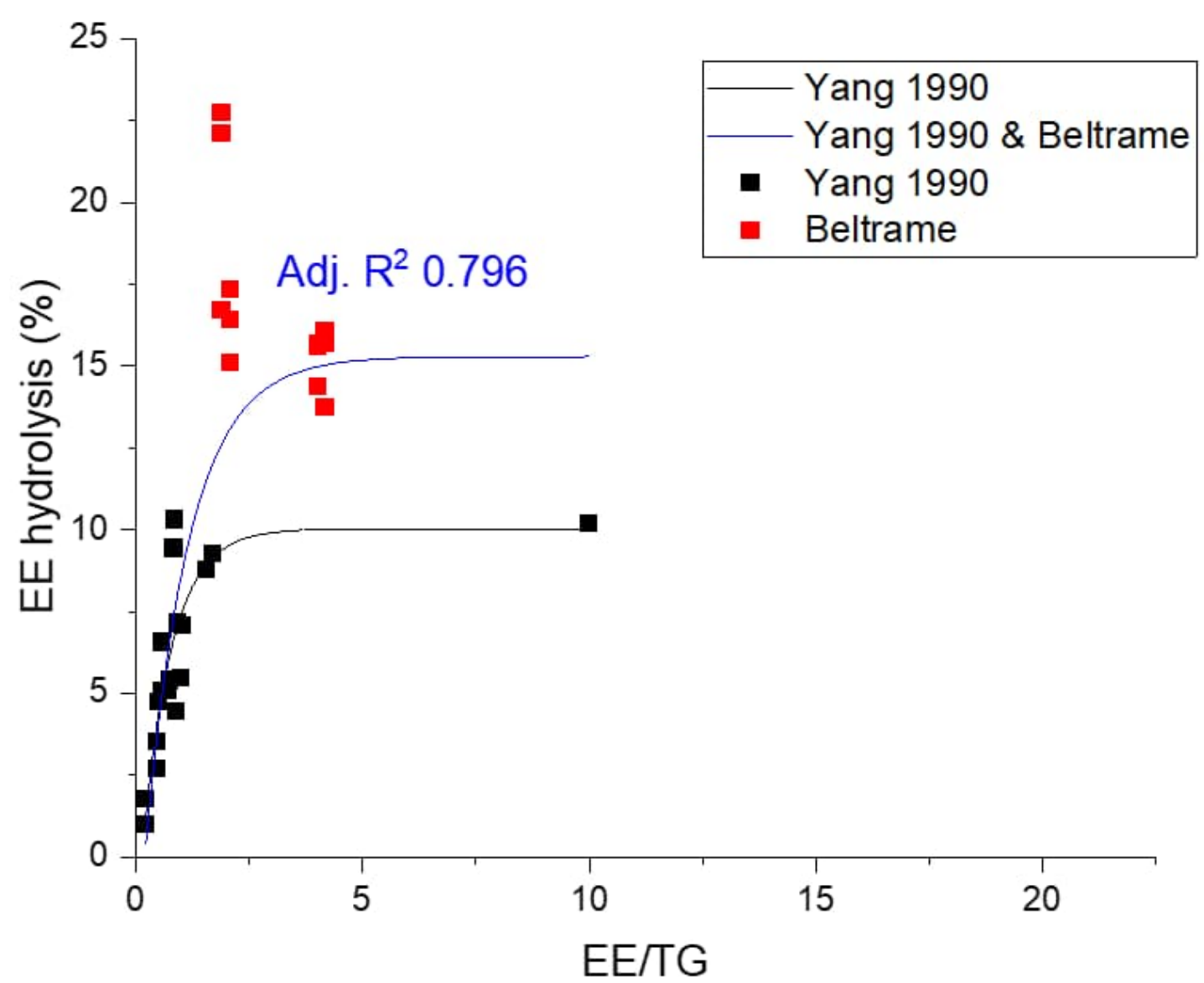

Supplementary Figure 2. Scatter plot comparing the present data with the report of Yang et al.[1] in terms of observed EE hydrolysis (%) after its digestion in presence of TAG and the corresponding EE-TAG ratio of lipids submitted to digestion.

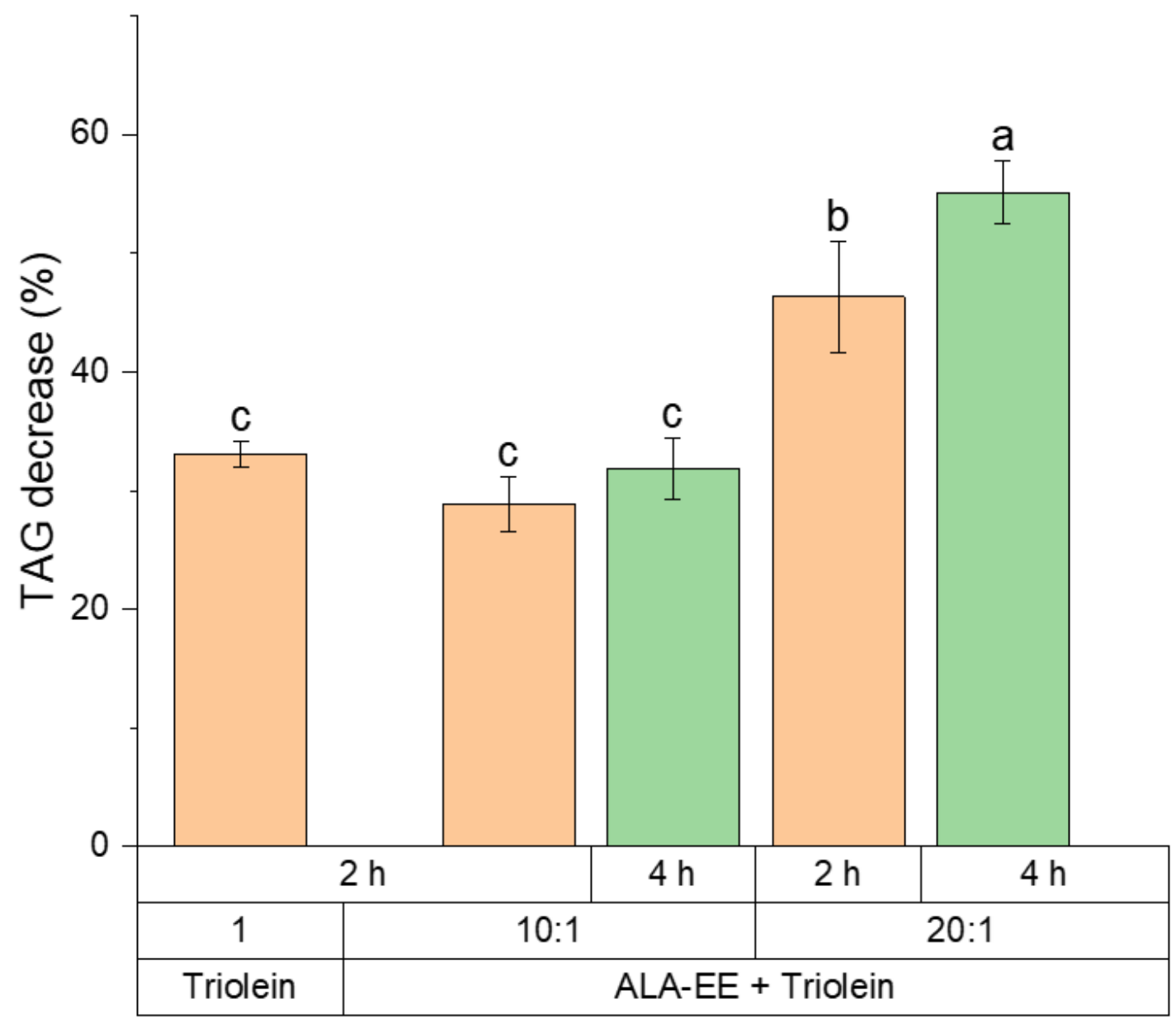

Supplementary Figure 3. Hydrolysis of triolein after simulated digestion represented as a decrease of total TAG signal in ^{1}H NMR spectra. Values are average ± standard deviation (n=3).

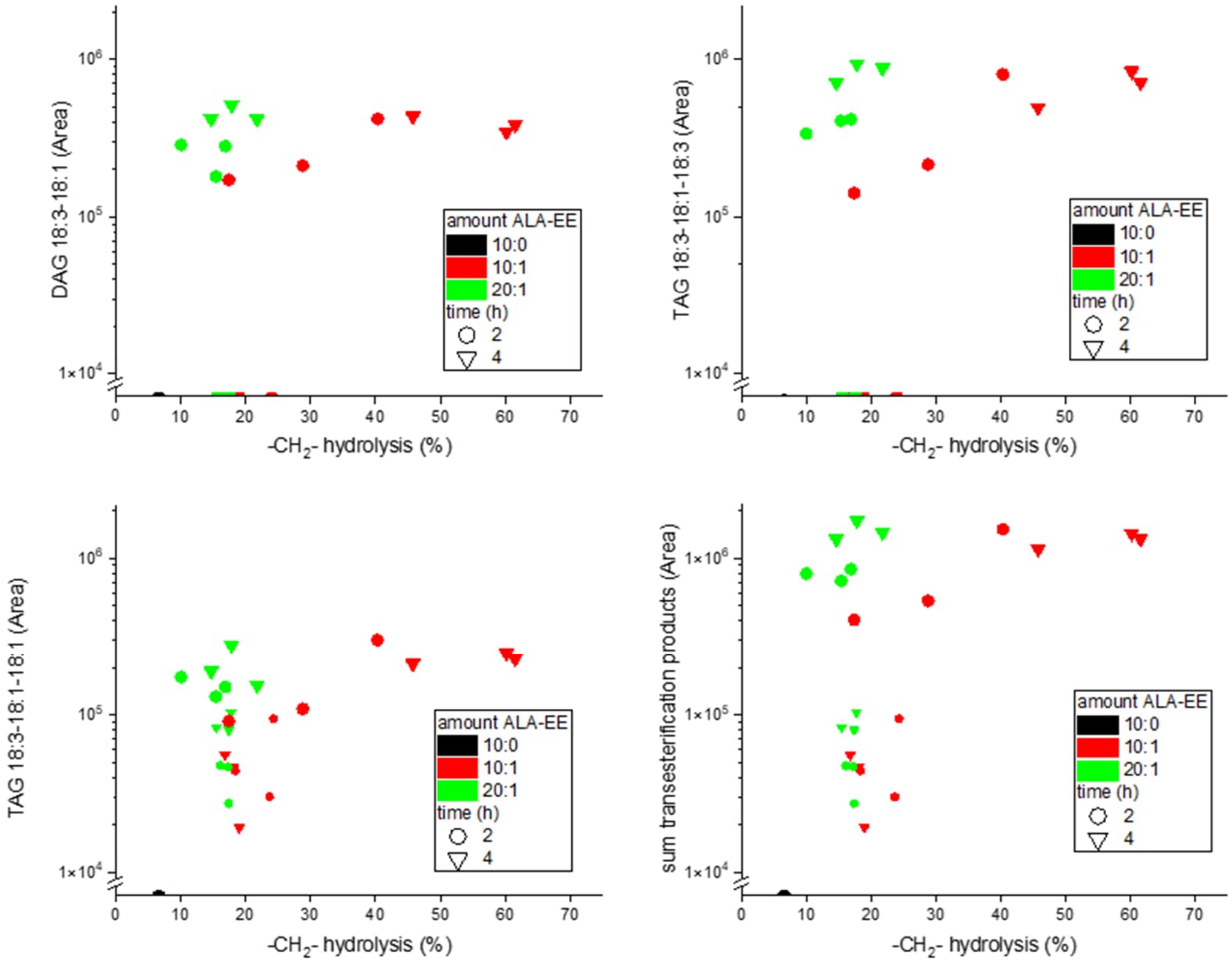

Supplementary Figure 4. Scattering plot showing the relationship between UHPLC-ESI-QTOF peak areas of transesterification products (DAG 18:3-18:1, TAG 18:3-18:1-18:1, TAG 18:3-18:1-18:3, and their relative sum) and hydrolysis of ALA-EE analyzed with ^{1}H NMR spectra in the respective digesta. Digesta containing triolein and 2-monoolein are distinguished by the use of smaller and larger markers, respectively.

Supplementary Table 1. Different parameters used in INFOGEST simulated digestion; intestinal phase duration and weight-based ratio of ALA-EE to triolein and 2-monoolein.

Ratio (w-w)			Intestinal phase duration (h)
**ALA-EE**	**2-monoolein**	**Triolein (Estimated 2-MAG amount after digestion)**	
10	0	0	2
10	1	0	2
10	1	0	4
20	1	0	2
20	1	0	4
10	0	5.3 (1)	2
10	0	4.8 (1)	4
20	0	5.0 (1)	2
20	0	4.8 (1)	4

Supplementary Table 2. Chemical shift assignments and multiplicities of the ^{1}H NMR signals of the protons present in acylglycerols, ethyl ester and fatty acids of samples before and after in vitro digestion. The signal letters correspond to ones given in figure 6. Abbreviations: t = triplet, m = multiplet, d = doublet, q = quartet.

Signal	Chemical shift (ppm)	Multiplicity	Type of protons	Compound
A	0.88	t	$\text{-C}\mathbf{H}_3$	Saturated, monounsaturated n-9 acyl groups and FA
B	0.97	t	$\text{-C}\mathbf{H}_3$	Unsaturated n-3 acyl groups and FA
C	1.24–1.38	m	$\text{-(C}\mathbf{H}_2\text{)}_n\text{-}$	Acyl groups and FA
D	1.62	m	$\text{-OCO-CH}_2\text{-C}\mathbf{H}_2\text{-}$<br><br>$\text{COOH-CH}_2\text{-C}\mathbf{H}_2\text{-}$	Acyl groups and FA
E	1.98–2.11	m	$\text{-C}\mathbf{H}_2\text{-CH=CH-}$	Acyl groups and FA
F	2.29–2.38	dt<br>m<br>t<br><br>t	$\text{-OCO-C}\mathbf{H}_2\text{-}$<br>$\text{-OCO-C}\mathbf{H}_2\text{-}$<br>$\text{-OCO-C}\mathbf{H}_2\text{-}$<br>$\text{COOH-C}\mathbf{H}_2\text{-}$<br>$\text{-OCO-C}\mathbf{H}_2\text{-}$	Acyl groups in TAG<br>Acyl groups in 1,2-DAG<br>Acyl groups in 1-MAG and FA<br>Acyl groups in 2-MAG
G	2.81	m	$\text{=HC-C}\mathbf{H}_2\text{-CH=}$	Polyunsaturated n-3 acyl groups and FA
H	3.73	dd	$\text{ROCH}_2\text{-CH(OR')-C}\mathbf{H}_2\text{OH}$	Glyceryl group in 1,2-DAG
I	3.84	dd	$\text{HOC}\mathbf{H}_2\text{-CH(OR)-C}\mathbf{H}_2\text{OH}$	Glyceryl group in 2-MAG

Signal	Chemical shift (ppm)	Multiplicity	Type of protons	Compound
J	3.97	m	$ROCH_2$-C**H**OH-CH_2OH	Glyceryl group in 1-MAG
K	4.12	q	CH_3–C**H₂**–OCOR	Methylene group in EE
L	4.24	ddd	ROC**H₂**-CHOH-CH_2OH	Glyceryl group in 1-MAG
M	4.15, 4.29	dd,dd	ROC**H₂**-CH(OR')-CH_2OR''	Glyceryl group in TAG
N	4.33	ddd	ROC**H₂**-CH(OR')-CH_2OH	Glyceryl group in 1,2-DAG
O	4.93	m	$HOCH_2$-C**H**(OR)-CH_2OH	Glyceryl group in 2-MAG
P	5.08	m	$ROCH_2$–C**H**(OR')-CH_2OH	Glyceryl group in 1,2-DAG
Q	5.26	m	$ROCH_2$–C**H**(OR')-CH_2OR''	Glyceryl group in TAG
R	5.28–5.43	m	–C**H**=C**H**–	Acyl groups and FA

Supplementary Table 3. Ratios between sum of transesterification products (DAG 18:3-18:1, TAG 18:3-18:1-18:1 and TAG 18:3-18:1-18:3) and 2-monoolein after INFOGEST simulated digestion quantified with UHPLC-ESI-QTOF and ^{1}H NMR data. Values are average ± standard deviation (n=3). Letters mark statistically significant differences between samples ($p < 0.05$).

Sample	Transesterification products : 2-monoolein ratio	
	**UHPLC-ESI-QTOF**	**^{1}H NMR**
ALA-EE + 2-monoolein 10:1 2h	0.90 ± 0.64a	0.39 ± 0.23a
ALA-EE + 2-monoolein 10:1 4h	2.14 ± 0.28a	1.06 ± 0.10b
ALA-EE + 2-monoolein 20:1 2h	0.99 ± 0.17a	0.45 ± 0.12a
ALA-EE + 2-monoolein 20:1 4h	2.28 ± 0.51a	0.73 ± 0.08a,b

References

(1) Yang, L.-Y.; Kuksis, A.; Myher, J. J. Intestinal Absorption of Menhaden and Rapeseed Oils and Their Fatty Acid Methyl and Ethyl Esters in the Rat. *Biochem. Cell Biol.* **1990**, *68* (2), 480–491. https:--doi.org-10.1139-o90-068.
